# The association of different target temperatures in targeted temperature management with neurological outcome after out-of-hospital cardiac arrest based on a prospective multicenter observational study in Korea (the KORHN-PRO registry): IPTW analysis

**DOI:** 10.1371/journal.pone.0271605

**Published:** 2022-07-22

**Authors:** Hyo Joon Kim, Chun Song Youn, Kyu Nam Park, Young-Min Kim, Byung Kook Lee, Kyung Woon Jeung, Won Young Kim, Seung Pill Choi, Soo Hyun Kim, on behalf of the Korean Hypothermia Network Investigator

**Affiliations:** 1 Department of Emergency Medicine, Seoul St. Mary Hospital, College of Medicine, College of Medicine, The Catholic University of Korea, Seoul, South Korea; 2 Department of Emergency Medicine, Chonnam National University Hospital, Chonnam National University Medical School, Donggu, Gwangju, Republic of Korea; 3 Department of Emergency Medicine, Asan Medical Center, University of Ulsan College of Medicine, Seoul, Republic of Korea; 4 Department of Emergency Medicine, Eunpyeong St. Mary Hospital, College of Medicine, The Catholic University of Korea, Seoul, South Korea; Stony Brook University Renaissance School of Medicine, UNITED STATES

## Abstract

**Background:**

Among comatose survivors of out-of-hospital cardiac arrest (OHCA), targeted temperature management (TTM) has improved neurological outcomes. However, although the target temperature shifted from 33°C to 33°C~36°C, the optimal target temperature is still unclear. The goal of this study was to evaluate neurological outcomes at 6 months at target temperatures of 33°C and 36°C.

**Materials and methods:**

We analyzed OHCA survivors who underwent TTM and were recorded in the Korean Hypothermia Network, a prospective multicenter registry, from October 2015 to December 2018. The primary outcome was good neurological outcome at six months, defined as a cerebral performance category of 1–2, and the secondary outcome was survival at 6 months.

**Results:**

A total of 1339 patients were treated with TTM in twenty-two emergency departments. Of those, 1054 were treated at 33°C, and 285 were treated at 36°C. There was no significant difference in good neurological outcomes at 6 months (30.6% vs. 31.2%, p = 0.850, adjusted OR 0.97, 95% CI = 0.73–1.29]) and survival at six months (41.4% vs. 38.7%, p = 0.401, adjusted HR 1.08, 95% CI = 0.91–1.28]) between TTM 33°C and TTM 36°C. After propensity score matching, good neurological outcomes at 6 months (OR 0.93, 95% CI = 0.74–1.18) and survival at 6 months (HR 1.05, 95% CI = 0.92–1.21) were still not associated with TTM 33°C and TTM 36°C.

**Conclusion:**

In this study, patients treated with a target temperature of 33°C had similar good neurological outcomes and survival at six months compared with those treated with a target temperature of 36°C.

## Introduction

Despite recent advances in critical care for patients with postcardiac arrest syndrome (PCAS), hypoxic-ischemic brain injury (HIBI) is common, impairs functional recovery, and ultimately results in in-hospital death due to failure to awaken [1]. Targeted temperature management (TTM) is the only therapeutic intervention to mitigate HIBI after cardiac arrest (CA) and is recognized as standard care for PCAS patients [2].

After two landmark studies were published in 2002, international guidelines recommended TTM for out-of-hospital cardiac arrest (OHCA) patients [3, 4]. However, recently published large-scale randomized studies reported conflicting results, with different effects of TTM depending on the characteristics of the included patients. A targeted temperature management (TTM) trial reported in 2013 demonstrated that TTM at 33°C was not superior to TTM at 36°C in OHCA patients with a presumed cardiac cause [5]. The use of TTM decreased significantly, which was associated with tendencies toward worse outcomes and a higher occurrence of fever after publication of the TTM trial [6, 7]. On the other hand, a trial published in 2019 found that TTM at 33°C was superior to controlled normothermia in patients with nonshockable rhythm [8]. This means that the optimal target temperature may differ depending on the epidemiological characteristics of PCAS patients, and this is due to the heterogeneity of PCAS patients. The conflicting study results have caused confusion for clinicians and require resolution. One recent study reported that TTM at 33°C improved survival in patients with the most severe postcardiac arrest illness without severe brain edema or malignant EEG and that TTM at 36°C was associated with better survival in mild to moderate patients [9]. Therefore, the optimal target temperature should be tested in various population groups.

We tested the hypothesis that the effect of TTM will be different in populations with different epidemiologic characteristics. Therefore, we aimed to evaluate the association between different target temperatures and long-term neurological outcomes in a prospective observational registry from Korea.

## Methods

### Study design

This study was a prospective, multicenter, observational cohort study. The Korean Hypothermia Network (KORHN) is a multicenter clinical research consortium for TTM in South Korea, and the Korean Hypothermia Network prospective registry (KORHN-PRO 1.0) is a web-based registry of OHCA patients treated with TTM between October 2015 and December 2018. The twenty-two academic hospitals that participated in the KORHN-PRO were required to fill out the participation form provided on the website of the KORHN-PRO registry (http://pro.korhn.or.kr/), which collected 136 variables with 839 datasets, and the participating institutions were distributed evenly across the country. The study included an informed consent form approved by the Institutional Review Boards (IRB) of all participating hospitals, including the IRB of Seoul St. Mary’s Hospital (XC15OIMI0081K) and the study was registered on the International Clinical Trials Registry Platform (NCT02827422) [10]. Written informed consent was obtained from all patients’ legal surrogates.

### Population

Adult patients with nontraumatic cardiac arrest who were treated with TTM were included in this study. For this study, we grouped patients according to their intended regimen. Patients planning treatment with a target temperature setting of 32–34°C were considered the TTM 33 group, and patients planning treatment with a target temperature setting of 34–36°C were considered the TTM 36 group. Patients with active intracranial bleeding, acute stroke, known limitations in therapy and a do-not-attempt resuscitation order, known prearrest cerebral performance category (CPC) 3 or 4, body temperature of 30°C on admission and unknown outcomes for 6 months after return of spontaneous circulation (ROSC) were excluded. The TTM protocol, including the core temperature setting, TTM duration, and TTM methods, was dependent on following the protocol [11]. The study outcome was a good neurological outcome at 6 months after ROSC, defined as a cerebral performance category (CPC) 1 and 2. The CPC scale ranges from 1 to 5: 1 represents good cerebral performance or slight cerebral disability, 2 represents moderate disability or independent activities of daily life, 3 represents severe disability or dependence on others for daily support, 4 represents a coma or vegetative state, and 5 represents death or brain death.

### Data collection

We extracted demographic data from the registry, such as age, sex, comorbidities, witnessed arrest, bystander CPR, and time from collapse to ROSC. To compare the severity of initial injury, we calculated the OHCA score using registry data [12]. During the TTM period, we recorded the following data prospectively: target temperature, target duration, temperature monitoring site, hourly body temperature from ROSC to 96 hours, TTM method, and time from ROSC to start TTM.

To evaluate adverse events, we collected the following data within 7 days after ROSC: seizures, bleeding, infections, sepsis, pneumonia, hypokalemia, hypoglycemia [blood glucose < 60 mg/dL], sustained hyperglycemia [blood glucose > 180 mg/dL for > 4 hours], tachycardia [> 130/min], and bradycardia [< 40/min]), and the rearrest events were documented.

Neurologic outcomes were investigated by the researchers from each hospital and evaluated at discharge and 1 and 6 months after cardiac arrest. The researcher contacted surviving discharged patients or their relatives. Follow-up was recommended at a face-to-face visit or by telephone interviews [11, 13].

### Statistical analysis

Categorical variables are presented as counts and percentages, and continuous data are presented as the mean ± standard deviation (SD). To compare differences in patient characteristics and outcomes, t tests, Fisher’s exact test and the chi-square test were used. We tested the distribution of continuous variables for mortality using visual inspection and the Shapiro–Wilk test.

We also performed multivariate logistic regression to investigate the interaction across the TTM target temperature range to achieve good neurological outcomes. To address characteristic differences, we calculated propensity scores by multivariate logistic regression with TTM treatment. These propensity scores were used to apply the inverse probability of treatment weights (IPTW) to generate a pseudostudy cohort. This weighted version can be balanced against the covariate bias and mimics the situation of random treatment assignments: the IPTW weights for TTM 33-treated patients = 1/p(treated) and for TTM 36-treated patients = 1/[1-p(treated)]. All analyses were performed in a univariate and multivariate manner before and after inverse probability of treatment weights.

All statistical analyses were performed using SAS version 9.4 (SAS Institute, Inc., Cary, NC, USA) and R version 4.0.3, and a p value <0.05 was considered statistically significant.

## Result

**Fig 1:**
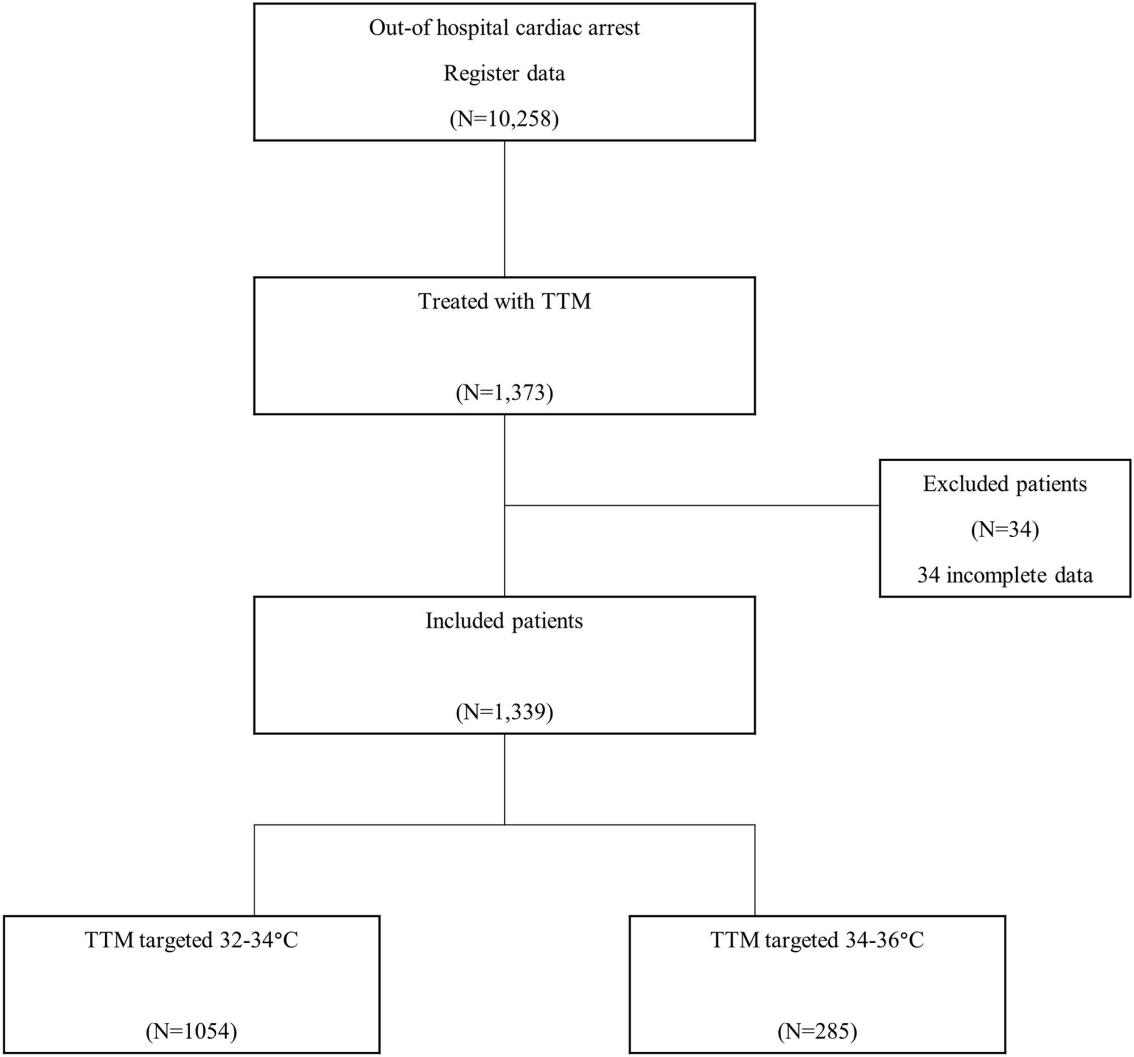
Flow-chart for study patients with out-of-hospital cardiac arrest between October 2015 and December 2018.

Between October 2015 and December 2018, a total of 10,258 OHCA patients were first screened for enrollment. Of those, 1,373 were treated with TTM, and 1,339 patients with complete data, including the 6-month neurological outcomes, were eligible for the final analysis (Fig 1).

A total of 961 patients (71.0%) were male, and the mean age was 58 years old. There were 839 patients (62.0%) with presumed cardiac etiology, 937 (69.2%) patients with witnessed arrest, 834 patients (61.6%) who received bystander CPR, and 482 patients (35.6%) with initially shockable rhythm.

The baseline characteristics of TTM 33 and TTM 36 are shown in Table 1. Compared to the TTM 36 group, the TTM 33 group patients had higher frequencies of witnessed arrest and higher lactic acid levels before the onset of TTM. To compare the initial severity of patients, we calculated the OHCA score in both groups, and the TTM 36 group had a higher OHCA score than the TTM 33 group.

**Table 1:** Baseline characteristics of OHCA patients.

Variables	TTM (33°C)	TTM (36°C)	*P* value
	N = 1054	N = 285	
Age	57.78 ± 15.55	58.76 ± 16.49	0.352
Sex (male, %)	758 (71.9)	194 (68.1)	0.204
Witnessed arrest (%)	754 (71.5)	175 (61.4)	0.001
Bystander CPR (%)	636 (60.3)	186 (65.3)	0.130
Initial shockable rhythm (%)	372 (35.3)	104 (36.5)	0.708
Past history			
HTN (%)	383 (36.3)	98 (34.4)	0.542
DM (%)	264 (25.1)	62 (21.8)	0.250
Acute MI (%)	73(6.8)	14(4.7)	0.206
Chronic heart failure	32(3.0)	19(6.4)	0.005
Previous PCI	39(3.6)	12(4.1)	0.717
CABG	14(1.3)	1(0.3)	0.160
CVA	52(4.8)	17(5.8)	0.513
Pulmonary disease	75(7.0)	19(6.8)	0.756
Renal disease	82(7.6)	22(7.5)	0.932
Liver cirrhosis	14(1.3)	8(2.7)	0.087
Collapse to ROSC interval (min)	27.01 ± 14.47	26.94 ± 15.19	0.945
Cardiac cause of arrest (%)	665 (63.1)	164 (57.5)	0.087
Clinical characteristics on admission			
Arterial pH	7.07 ± 0.20	7.06 ± 0.18	0.394
Lactate, mmol/L[IQR]	9.50[5.80–12.90]	10.35[7.25–13.50]	0.015
Epinephrine dose (mg), [IQR]	2[0–3]	2[0–4]	0.361
Temperature, [IQR]	35.7[35.3–36.3]	35.6[35.3–36.3]	0.421
Mean arterial pressure	92.33±31.84	90.19±31.54	0.315
GCS-M1 no. (%)	807(77.8)	221(75.4)	0.388
OHCA score	35.92 ± 18.25	38.65 ± 17.86	0.032

*Abbreviations*: *IQR* interquartile range *HTN* hypertension, *DM* diabetes mellitus, *Acute MI* acute myocadiac infraction, *PCI* percutaneous coronary intervention, *CABG* coronary artery bypass grafting, *CVA* cerebrovascular accident, *ROSC* Return of spontaneous circulation, *GCS-M1* Glasgow coma scale-motor grade 1

**Fig 2:**
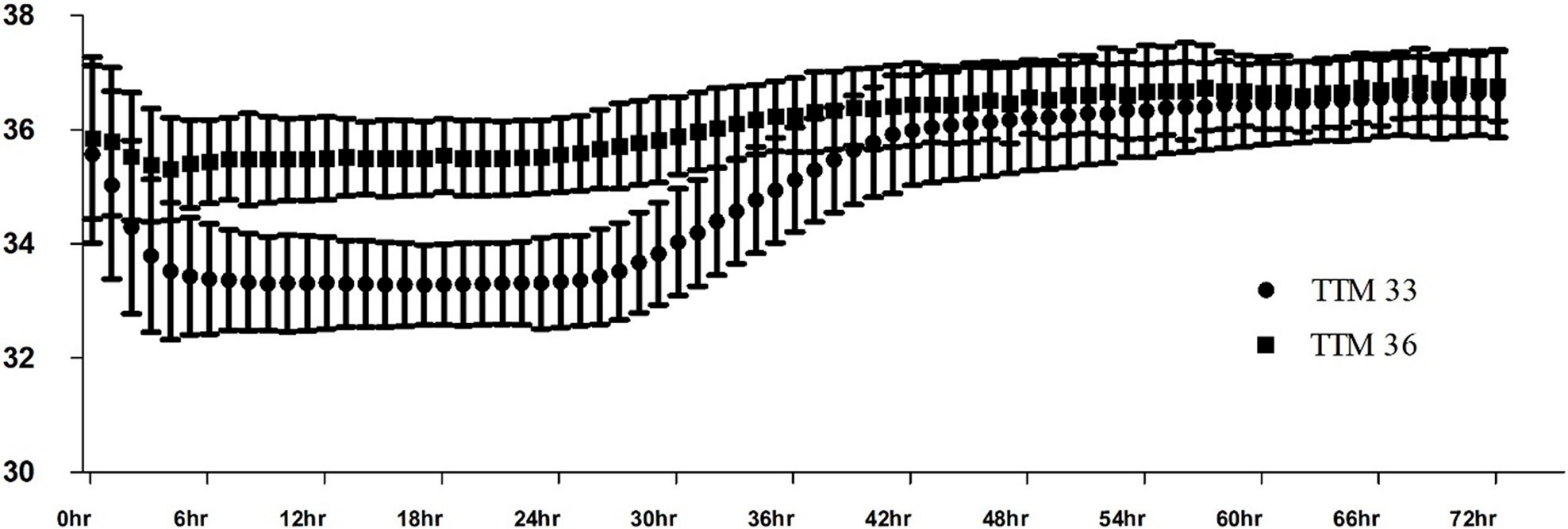
Temperatures in the study groups until 72 hours after target ROSC. Values are presented as the mean ±SDs.

TTM methods and intervention during TTM are shown in Table 2. The TTM 36 group used more surface cooling for the TTM method than the TTM 33 group; however, both groups mostly used the feedback method. Because the feedback device was used, body temperature was well controlled in both groups. The body temperature profile during the TTM periods is shown in Fig 2. During TTM, most patients received sedative agents and neuromuscular blockers, and the TTM 33 group had more coronary angiography (CAG) and thrombolysis; however, the percutaneous coronary angiography (PCI) rate was not significantly different between the two groups.

**Table 2:** TTM methods and intervention during TTM.

Variables	TTM (33°C)	TTM (36°C)	*P* value
	N = 1054	N = 285	
TTM method			
Feedback surface cooling	953(88.4)	269(91.2)	<0.001
Feedback endovascular cooling	121(11.2)	14(4.7)	
Others	4(0.4)	12(4.1)	
Sedative			
Midazolam	827(78.5)	181(63.5)	<0.001
Dexmedetomidine	137(12.9)	35(12.3)	0.708
Propofol	198(18.4)	35(12.3)	0.023
Neuromuscular blocker			
Atracurium	235(21.9)	14(4.7)	<0.001
Cisatracurium	228(21.2)	102(34.6)	<0.001
Rocuronium	279(25.9)	24(8.1)	<0.001
Vecuronium	171(15.9)	33(11.2)	0.085
Extracorporeal membrane oxygenation (ECMO)	42(3.9)	21(7.1)	0.019
Extracorporeal CPR	8(0.7)	7(2.4)	0.017
VA ECMO	33(3.1)	14(4.7)	0.159
VV ECMO	1(0.1)	0(0)	0.601
Intervention during ICU stay			
Coronary angiography (CAG)	378(35.1)	123(41.7)	0.036
PCI	153(14.2)	54(18.3)	0.080
Thrombolysis	41(3.8)	2(0.7)	0.023
ICD insertion	78(7.2)	18(6.1)	0.604
Mechanical circulatory assist			
IABP	7(0.7)	2(0.7)	0.857

*Abbreviations*: *PCI* percutaneous coronary intervention, *ICD* implantable cardioverter defibrillator, *IABP* intra-aortic balloon pump

Regarding complications during TTM, while bleeding was not associated with either TTM group, pneumonia, sepsis and rearrest were frequent in the TTM 33 group (Table 3).

**Table 3:** Complications during TTM and outcomes.

Variables	TTM (33°C)	TTM (36°C)	*P* value
	N = 1054	N = 285	
Complications during TTM			
Bleeding	50(4.6)	15(5.1)	0.749
Major bleeding	7(0.6)	2(0.7)	0.957
Critical organ bleeding	18(1.7)	3(1.0)	0.418
Other significant bleeding	25(2.3)	10(3.4)	0.301
Pneumonia	437(40.5)	89(30.2)	<0.001
Sepsis	144(13.4)	17(5.8)	<0.001
Rearrest	218(20.2)	42(14.2)	0.020
Seizure	264(24.5)	64(21.7)	0.319
Hypokalemia	356(33.1)	95(32.2)	0.775
Hypoglycemia	128(11.9)	26(8.8)	0.139
Sustained hyperglycemia	547(50.7)	162(54.9)	0.204
Tachycardia >130/min	208(19.4)	71(24.1)	0.078
Bradycardia < 40/min	74(6.9)	22(7.5)	0.739
Renal replacement therapy	167(15.6)	56(19.1)	0.149
Length of stay in hospital	10[5.0–18.0]	12[6.0–21.5]	0.087
Survival to hospital discharge	593(55.0)	164(55.6)	0.858
In hospital death cause			
Cardiovascular cause	134(12.4)	28(9.5)	0.166
Cerebral cause	115(10.7)	47(15.9)	0.013
Multi organ failure	207(19.2)	46(15.6)	0.157
Good neurological outcomes at 6 months after ROSC	323(30.6)	89(31.2)	0.850
Survival at 6 months after ROSC	437(41.4)	111(38.7)	0.401

Abbreviations: *TTM* targeted temperature management, *ROSC* return of spontaneous circulation

### Neurological outcomes and mortality

The number of patients with good neurological outcomes at 6 months (CPC 1–2) was 323 (30.6%) in the TTM 33 group and 89 (31.2%) in the TTM 36 group. Survival at 6 months was also similar—437 (41.4%) in the TTM 33 group and 111(38.7%) in the TTM 36 group—while a cerebral cause was more common in the TTM 33 group for in-hospital death cause. Neurological outcomes and survival rates were not significantly different (p = 0.850, p = 0.410) (Table 3).

**Fig 3:**
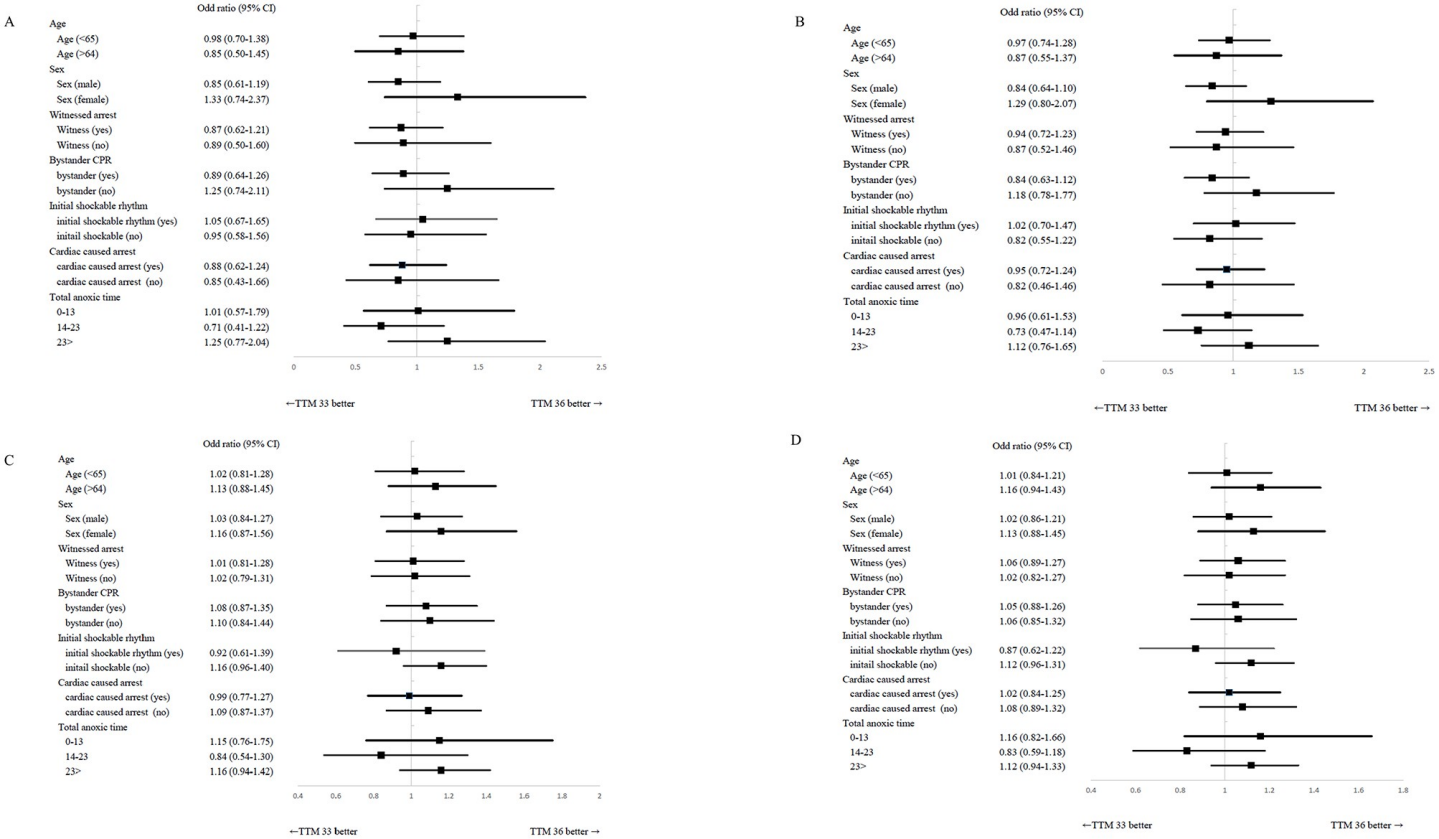
Forest plot for subgroup analysis of good neurological outcome and survival after six months from ROSC in patients treated with TTM analyzed with multivariate logistic regression and inverse probability treatment weighting(IPTW). A = Forest plot for subgroup analysis of good neurologic outcome after six months from ROSC in patients treated with TTM using multivariate analysis. B = Forest plot for subgroup analysis of good neurologic outcome after six months from ROSC in patients treated with TTM using IPTW. C = Forest plot for subgroup analysis of survival after six months from ROSC in patients treated with TTM using multivariate analysis. D = Forest plot for subgroup analysis of survival after six months from ROSC in patients treated with TTM using multivariate IPTW.

Age, sex, history of witnessed cardiac arrest, bystander CPR, initial shockable rhythm, cardiac cause of arrest, time from collapse to ROSC, and OHCA score were finally selected as covariates. When TTM 36 was included, multivariate analysis showed no statistically significant association with good neurological outcomes at 6 months (OR = 0.97, 95% CI 0.73–1.29), confirmed by inverse probability of treatment weighting (IPTW) (OR = 1.05, 95% CI = 0.92–1.21). Survival at 6 months was also not significantly different in multivariate analysis (HR = 1.08, 95% CI = 0.91–1.28) or IPTW (HR 1.05, 95% CI = 0.92–1.21) (Table 4). Subgroup analysis was performed according to 65 years of age, sex, presence of witnessed arrest, presence of bystander CPR, presence of initial shockable rhythm, presence of cardiac-caused arrest, and total anoxic time. However, both survival and good neurological outcomes were not different between the TTM 33 group and the TTM 36 group in multivariate logistic regression and IPTW analysis (Fig 3).

**Table 4:** Odds ratio for neurological outcomes and survival at 6 months after ROSC among patients treated with TTM at 36°C.

	OR (95% CI)	p Value
*Multivariate logistic regression*		
Good neurological outcomes at 6 months after ROSC	0.97(0.73–1.29)	0.850
Survival at 6 months after ROSC	1.08(0.91–1.28)	0.375
*Propensity score matched*		
Good neurological outcomes at 6 months after ROSC	0.93(0.74–1.18)	0.557
Survival at 6 months after ROSC	1.05(0.92–1.21)	0.465

Adjusted for age, sex, witnessed arrest, bystander CPR, initial shockable rhythm, cardiac cause of arrest, time from collapse to ROSC, and OHCA score

## Discussion

In this large-scale prospective, multicenter, observational registry study, we analyzed out-of-hospital cardiac arrest patients treated with TTM33 and TTM36. The main findings of this study were that there was no statistically significant difference in neurological outcomes or survival rate at 6 months after ROSC.

In our study, the most common target temperature was 33°C. Although the proportion of patients with nonshockable rhythm was high in this study, the neurological prognosis was not significantly different from that of other studies. In an earlier TTM trial, 46% of the patients in the TTM 33 group and 48% of the patients in the TTM 36 group had good neurological outcomes, whereas in this study, 64.5% of the patients with initial shockable rhythm in the TTM 33 group and 63.5% of those in the TTM 36 group had good neurological outcomes [5]. Among patients with nonshockable rhythm, a recent study showed that 10.2% of patients in the TTM 33 group and 5.7% of patients in the normothermia group had good neurological outcomes, and the current study showed that 12.2% of patients in the TTM 33 group and 12.7% of patients in the TTM 36 group had good neurological outcomes [8]. This may be due to advances in intensive care, and it can be thought that the patients this registry were well controlled regarding temperature using feedback devices for almost all patients.

Although previous studies have attempted to evaluate the optimal core temperature, the results have been controversial. Earlier studies have shown high fever and mortality rates when treated with TTM 36°C rather than TTM 33 [6, 7]. In this registry, although a target temperature of 33°C was the most common, the proportion of patients with a target temperature of 36°C has been increasing over the years. Even in the group treated with a target temperature at 36°C, it was very rare to have a fever of more than 37°C using most of the feedback devices. This is presumed to be the reason that the proportion of patients treated at 36°C increases as the year goes by, and the poor prognosis does not increase compared to those treated at 33°C.

A recent TTM 2 trial reported the difference between targeted hypothermia at 33°C and targeted normothermia with early treatment of fever [14]. According to this study, there was no significant difference in the functional outcome and mortality between the 33°C targeted hypothermia and the early treatment of fever group. Although the rate of nonshockable rhythm increased compared to the previous TTM trail, the rate of shockable rhythm was still higher than that of this study. Also, in the normothermia group, 46%f of patients controlled body temperature with a cooling device. As in this study, when the ratio of non-shockable rhythm is high, it is thought that more study is needed on how much cooling device is needed and how the neurologic outcome is different.

Recently, several studies have found optimal target temperatures according to the initial severity. Callaway *et al*. showed that TTM at 33°C was associated with better outcomes than TTM at 36°C among patients with the most severe post–cardiac arrest illness but without severe cerebral edema or malignant EEG [9]. Using rCAST risk stratification tools, M. Nishikimi *et al*. reported that moderate-severity patients were associated with good neurological outcomes when treated with a target temperature at 33–34°C [15]. In this study, we analyzed subgroups to identify the characteristics of patients with a good neurological prognosis for a specific body temperature but could not reach a conclusion. This may be because the proportion of patients treated with TTM at 36°C is low and the choice of key temperature settings for TTM is left to the discretion of each participating hospital. It is also thought that this is because patients with shock after ROSC and patients with high OHCA scores were treated with TTM at 36°C more often. Despite these points, registry data should be considered as well since registry data can better reflect reality.

Target temperature management is helpful for neurological outcomes, but a decrease in body temperature can potentially increase the risk of complications. The known complications are an increased risk of infection due to immunosuppression, hypotension, electrolyte disturbance, and coagulopathy [16]. Kleissner M. *et al*. reported more major bleeding and hypotension with target temperature management at 34–36°C [17].

Although there were more patients treated with extracorporeal membrane oxygenation, Kagawa *et al*. reported more complications with treatment at < 34°C [18]. In this study, there were no significant differences in bleeding complications between TTM 33 and TTM 36, but more infections and rearrangements occurred at TTM 33. This is probably due to the high severity of the initial injury due to the high number of patients with unwitnessed arrest and nonshockable rhythms in our study. Therapeutic hypothermia often results in hypothermia-induced electrolyte imbalance due to cold diuresis [19], and in a TTM trial, there was more hypokalemia in the TTM 33 group [5]. In this registry, the hypokalemia proportion was greater than that in the TTM trial, and the TTM 33 group had a higher incidence of hypokalemia; however, there was no significant difference, and more research on this is needed.

There are several limitations to our study. First, although the data manager monitored the data, there were some missing data in the KORHN-PRO registry, which might have affected the results. Second, most of the included hospitals were teaching or university-affiliated hospitals located within the nation’s capital region, and the choice of the target temperature management setting was dependent on the preference at each participating site. The existence of selection bias or confounding variables cannot be completely excluded. Third, during the study period, the number of TTM 36 patients increased every year, but the proportion of TTM 36 patients was still low, which may affect outcomes.

## Conclusions

In conclusion, in this large multicenter, prospective registry study of OHCA patients treated with targeted temperature management, there were no significant differences in good neurological outcomes or survival rates 6 months after ROSC between TTM 33 and TTM 36.

## Supporting information

S1 Data Data of KORHN prospective registry. (PDF)
